# A detailed method for preparation of a functional and flexible blood–brain barrier model using porcine brain endothelial cells^☆^

**DOI:** 10.1016/j.brainres.2013.04.006

**Published:** 2013-07-12

**Authors:** Adjanie Patabendige, Robert A. Skinner, Louise Morgan, N. Joan Abbott

**Affiliations:** a Institute of Pharmaceutical Science, Franklin Wilkins Building, King's College London, London, UK; b Faculty of Life Sciences, University of Manchester, Manchester, UK; c Eisai Limited, European Knowledge Centre, Hatfield, UK

**Keywords:** Blood–brain barrier, Brain endothelium, *In vitro* model, Transendothelial electrical resistance, Tight junction, Permeability

## Abstract

The blood–brain barrier (BBB) is formed by the endothelial cells of cerebral microvessels and forms the critical interface regulating molecular flux between blood and brain. It contributes to homoeostasis of the microenvironment of the central nervous system and protection from pathogens and toxins. Key features of the BBB phenotype are presence of complex intercellular tight junctions giving a high transendothelial electrical resistance (TEER), and strongly polarised (apical:basal) localisation of transporters and receptors. *In vitro* BBB models have been developed from primary culture of brain endothelial cells of several mammalian species, but most require exposure to astrocytic factors to maintain the BBB phenotype. Other limitations include complicated procedures for isolation, poor yield and batch-to-batch variability. Some immortalised brain endothelial cell models have proved useful for transport studies but most lack certain BBB features and have low TEER. We have developed an *in vitro* BBB model using primary cultured porcine brain endothelial cells (PBECs) which is relatively simple to prepare, robust, and reliably gives high TEER (mean∼800 Ω cm^2^); it also shows good functional expression of key tight junction proteins, transporters, receptors and enzymes. The model can be used either in monoculture, for studies of molecular flux including permeability screening, or in co-culture with astrocytes when certain specialised features (e.g. receptor-mediated transcytosis) need to be maximally expressed. It is also suitable for a range of studies of cell:cell interaction in normal physiology and in pathology. The method for isolating and growing the PBECs is given in detail to facilitate adoption of the model.

*This article is part of a Special Issue entitled Companion Paper*.

## Highlights

• We have given a detailed method for preparation of an **in vitro** porcine BBB model.
• The model is functionally characterised for major BBB features.
• The monocultured model gives high transendothelial electrical resistance.
• The monocultured model is suitable for BBB drug permeability screening.
• Co-culture with astrocytes can be used if needed to enhance specific BBB features.

*This article is part of a Special Issue entitled Companion Paper*.

*This article is part of a Special Issue entitled Companion Paper*.

*This article is part of a Special Issue entitled Companion Paper*.

## 1 Introduction

The blood–brain barrier (BBB) is formed by the endothelial cells of cerebral microvessels under the influence of associated cells of the neurovascular unit (NVU), chiefly pericytes and the end-feet of perivascular astrocytes (Abbott et al., 2006; Neuwelt et al., 2011; Wolburg et al., 2009). The BBB is the protective interface regulating molecular, ionic and cellular traffic between the blood and the central nervous system (CNS). The barrier has several key features (Abbott et al., 2010). The ‘physical barrier’ results from the nature of the lipid membranes and presence of particularly tight intercellular *zonulae occludentes* (tight junctions); the junctions help to segregate apical and basal membrane proteins, conferring strong cellular polarity, and significantly restrict permeability of small hydrophilic solutes through the intercellular cleft (paracellular pathway), giving rise to the high transendothelial electrical resistance (TEER) (Abbott et al., 2010; Tsukita et al., 2001; Wolburg et al., 2009). The ‘transport barrier’ applies to transcellular flux of small and large molecules: solute transporter proteins (SLCs) and ATP-binding cassette (ABC) efflux transporters regulate traffic of small molecules (nutrients, substrates, waste products) (Begley, 2004; Mahringer et al., 2011; Miller, 2010), while specific vesicular mechanisms regulate permeation of peptides and proteins needed by the CNS (Bickel et al., 2001; Hervé et al., 2008; Jones and Shusta, 2007). The ‘enzymatic’ or ‘metabolic barrier’ function of the BBB results from the presence of a number of ecto- and endo-enzymes including cytochrome P450s (CYPs) that add a further level of protection (Ghosh et al., 2011). Finally the ‘immunological barrier’ restricts and regulates the entry of circulating leucocytes, maintaining a low level immune surveillance of the CNS, and with the potential for concerted response in conditions of pathology (Greenwood et al., 2011; Hawkins and Davis, 2005; Persidsky et al., 2006; Stanimirovic and Friedman, 2012).

*In vivo* studies continue to provide valuable information about the physiology and pathology of the BBB and operation of the NVU; however, for detailed molecular and functional understanding, *in vitro* models can give particular additional insights (Deli et al., 2005; Naik and Cucullo, 2012). Moreover, *in vitro* models allow rapid conduct of complex experiments involving parallel manipulation of bathing media, addition of inhibitors and calculation of transport kinetics while minimising the use of animals. For studies of transendothelial flux, including drug permeability assays, it is important to use models with well-developed tight junctions (high TEER) and well preserved apical:basal polarity of transporters and receptors (Abbott et al., 2008; Deli et al., 2005; Tóth et al., 2011).

The key features of the adult BBB result from a sequence of cell:cell interactions during development between the ingrowing vessel sprouts and the associated cells of the NVU (Liebner et al., 2011). When brain microvessels are isolated from adult mammalian brain and brain endothelial cells are cultured from these vessel fragments, they retain many key features of the BBB phe-notype. In 1969, Siakotos and colleagues described for the first time a method to successfully isolate bovine and human brain endothelial cells (Siakotos et al., 1969). Nearly a decade later, Panula et al. demonstrated the migration of rat brain endothelial cells from isolated capillaries. These cells were able to grow in culture and had strong alkaline phosphatase activity (Panula et al., 1978). Tontsch and Bauer (1989) simplified the culture methods for isolating murine and porcine brain endothelial cells (e.g. avoiding sieving steps, gradient centrifugations) and optimised the culture medium to increase cell yield. They also found that when proliferative factors such as endothelial cell growth supplement (ECGS) and heparin were removed from culture medium, the morphology of cells changed from spindle-shape to cobblestone phenotype. Through a series of experiments, DeBault and Cancilla gave evidence for the influence of astrocytic factors on BBB phenotype of brain endothelial cells (DeBault and Cancilla, 1980a, 1980b; DeBault, 1981). These studies led to the development of co-culture models of the BBB (Joó, 1985).

We chose to develop a porcine BBB model for several reasons: (1) A single pig brain gives a high yield of cells compared to that from rat or mouse. (2) Porcine brains are relatively easy to obtain as they are a by-product of the meat industry; there is no need to have animal breeding facilities on site to maintain a continuous supply of brain tissue. (3) Porcine brain endothelial cells (PBECs) generally retain many key features of the BBB following isolation, and the rate of loss of BBB phenotype in culture is less than for rodent or bovine BBB models (Deli et al., 2005), therefore co-culture with astrocytes is not essential to induce functional expression of tight junctions (i.e. high TEER) (Patabendige et al., this issue). (4) The porcine genome, anatomy, physiology and disease progression reflect human biology more closely than many established laboratory animals (Walters et al., 2011). (5) The availability of miniature pigs and novel porcine transgenic disease models make the pig the most suitable animal model to study human disease (Bendixen et al., 2010; Lunney, 2007). The miniature pig is now a well established ‘large’ mammalian model for pharmacokinetics/toxicology studies (Bode et al., 2010) and is also used for surgical studies to generate organs for xenotransplantation (Vodicka et al., 2005). Transgenic pig models have been established for stud-ying several diseases, including Alzheimer’s disease, Huntington's disease, cardiovascular disease, cystic fibrosis and diabetes mellitus (Aigner et al., 2010).

We have developed and validated a cell culture model of the BBB using PBECs with functional tight junctions (Patabendige et al., this issue). This model reliably gives high TEER (mean TEER∼800 Ω cm^2^) with good expression of tight junction proteins claudin-5, occludin and ZO-1, and shows expression of functional BBB transporters (P-glycoprotein, breast cancer-resistance protein), receptors (interleukin-1 receptor) and enzymes (alkaline phosphatase) (Patabendige et al., this issue; Skinner et al., 2009). The strengths of this model are that it is relatively simple and straightforward to generate compared to other published porcine BBB models and is able to give high TEER reliability even without co-culture with astrocytes. For certain specialised studies, BBB features can be further upregulated by exposure to astrocytes or astrocyte-conditioned medium (ACM). The model has been validated in studies of basic functions of the BBB at the cellular and molecular level, screening of drug entry into brain for pharmaceutical purposes, and examination of mechanism(s) for CNS entry of ‘biologicals’ (large organic molecules) (Patabendige et al., this issue; Skinner et al., 2009). It is highly suitable for a range of further studies including cell:cell interaction.

**Fig. 1:**
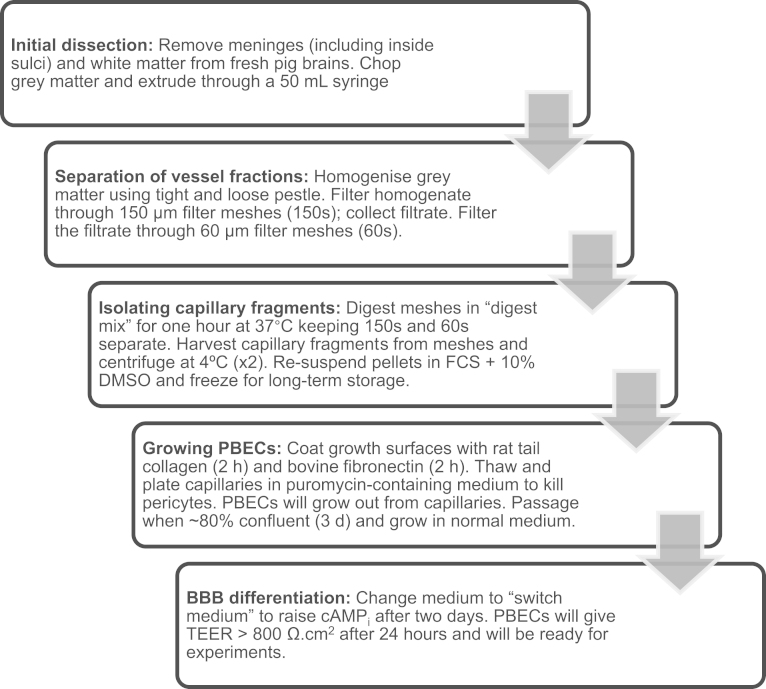
Flow chart showing porcine brain endothelial cell culture method (mono-culture variant). Sequence of procedure from dissection of pig brains to growth of porcine brain endothelial cells for experiments.

The aim of this paper is to give a detailed account of the method for isolation of porcine brain microvessels and culture of PBECs to establish a BBB model with high TEER. We present two variants of the model: (1) PBECs in monoculture—the simplest variant of the model which gives high TEER reliably (Fig. 1 summarises the method), and (2) PBECs co-cultured with rat astrocytes, useful when expression of a specific receptor, transporter, or vesicular transport system needs to be increased/induced using astrocytic factors. We have given a short history of the model, to show its development and refinement in three phases spanning over more than a decade of research. Optimal growing conditions for generating well-differentiated PBEC monolayers on plastic and on Transwell inserts for functional studies including examination of transendothelial solute flux were tested using different extracellular matrix coatings (type I collagen or rat tail collagen, with or without fibronectin), and elevation of intracellular cAMP (cAMP_i_). Both matrix composition and cAMP_i_ are known to affect the state of differentiation in a variety of cell types (Rubin et al., 1991; Tilling et al., 1998). To further encourage development of a BBB phenotype, we tested addition of hydrocortisone to improve tightness of the monolayer (Hoheisel et al., 1998), puromycin during early stages of growth to kill contaminating pericytes (Perrière et al., 2005) and addition of astrocyte factors (in ACM, or by co-culturing with astrocytes in a non-contact model) (Gaillard et al., 2001; Haseloff et al., 2005; Wolburg et al., 1994).

## 2 Results

### 2.1 Isolation of brain capillary endothelial cells

A homogeneous and smaller diameter vessel fraction was collected from the finer filters (60 µm mesh) than from the coarser filters (150 µm mesh). Furthermore, TEER of PBEC monolayers cultured from the 60 µm fraction was higher, consistent with the 60 µm fraction being derived from purer capillaries (60s: 625±21 Ω cm^2^, *n*=6, cf. 150s: 237±10 Ω cm^2^, *n*=6).

### 2.2 Characterisation

Characterisation of the brain endothelial cell monolayers produced by this method (Patabendige et al., this issue) and the co-culture variant (Skinner et al., 2009) are published elsewhere. By a range of morphological, immunocytochemical and functional criteria, the cells reproduce well *in vivo* endothelial and BBB features, from expression of endothelial markers, to organisation of tight junction proteins, and exp-ression of typical BBB enzymes and transport systems. They have been used for a number of studies on the cellular and molecular function of the BBB (in preparation).

### 2.3 Morphology, assessment of barrier integrity and reproducibility

TEER is one of the best measures of the barrier function of an *in vitro* BBB model, and has been used throughout the optimi-sation of this method and applications of the resulting model variants.

#### 2.3.1 Initial development of method

**Fig. 2:**
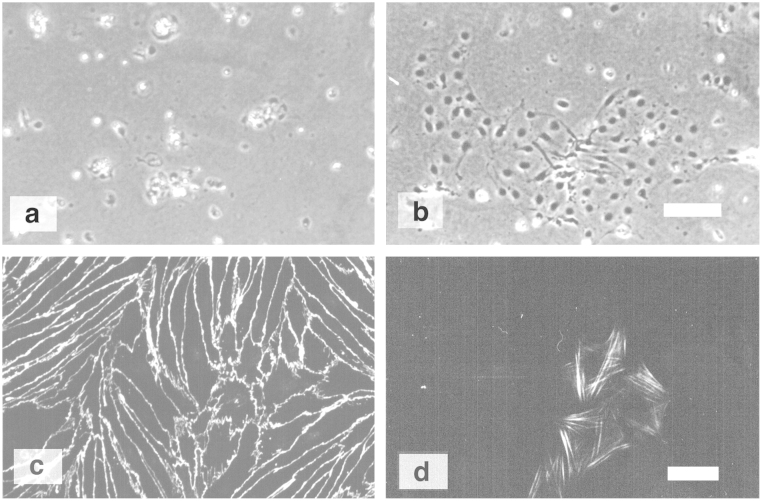
Porcine microvessel explants. Phase contrast micrograph of PBECs (a) 9 h, and (b) 3 days in culture. In 9 h the capillary fragments have attached to the collagen/fibronectin substrate and cell processes can be seen emerging from the explant. After longer in culture endothelial cells have migrated away from the explant site. To facilitate labelling, porcine microvessels were plated directly on to Transwell inserts and were grown for 5 days (c and d). The monolayer of PBECs, labelled with antibody to the adherens junction catenins p100/p120 (c), is continuous over the top of some cells that do not express endothelial cell markers, these cells label with antibodies to smooth muscle-specific actin (d). Scale bar: 100 µm in (a) and (b); 50 µm in (c) and (d).

The initial development of this method was carried out at Eisai laboratories (London), by modifying a protocol for bovine brain (Rubin et al., 1991). A primary aim was to keep the dissection and capillary isolation steps as short as possible, expecting that this would favour endothelial cell yield and viability. Hence although larger pieces of white matter and all of the meninges were removed in dissection, no fine cleaning to pick off small pieces of white matter was used. Capillary fragments were cultured in 50% ACM (with 10% bovine plasma-derived serum, BPDS):50% Dulbecco's modified Eagles medium (DMEM with 10% BPDS, 1% glutamine and 1% penicillin/streptomycin) and 125 µg/mL heparin. The cells took 4–5 days to reach 50–80% confluence and had a few contaminating cells, likely pericytes and connective tissue cells that labelled with antibodies against smooth muscle actin (Fig. 2). To generate a robust TEER, PBECs were established on Transwell filters in the growth medium (N2 defined medium with 10 µg/mL transferrin, 100 µM putrescine, 0.3 nM sodium selenite, 5 µg/mL insulin and 20 nM progesterone) containing 50% ACM and treated with agents that elevated cAMP_i_. Using this method, TEER in the range of 400–600 Ω cm^2^ could be obtained (Schulze et al., 1997), a 1.3–2.4-fold increase in TEER compared to cultures in ACM/N2 alone. To further increase TEER, passaged PBECs were also grown on Transwell filters in the growth medium containing 50% ACM in human endothelial serum-free medium (hESFM, Gibco), a formulation that contains hydrocortisone (Battista and Soderland, 1995; Gorfien et al., 1993). This caused a 2.5–3.5-fold increase in TEER compared to the cells in 50% ACM/N2 alone.

#### 2.3.2 Changes introduced to improve the quality of PBECs

Experience with a number of primary brain endothelial cell culture models in our groups and elsewhere has indicated that thorough removal of meninges and white matter, and treatment to kill pericytes lead to improvements in purity and yield, and in growth and barrier characteristics. Also, since the composition of Gibco hESFM used in the initial method is not reported in the literature, it appeared worthwhile to test simpler growth media (DMEM) supplemented with hydrocortisone.

#### 2.3.3 Influence of special treatments on barrier integrity

**Fig. 3:**
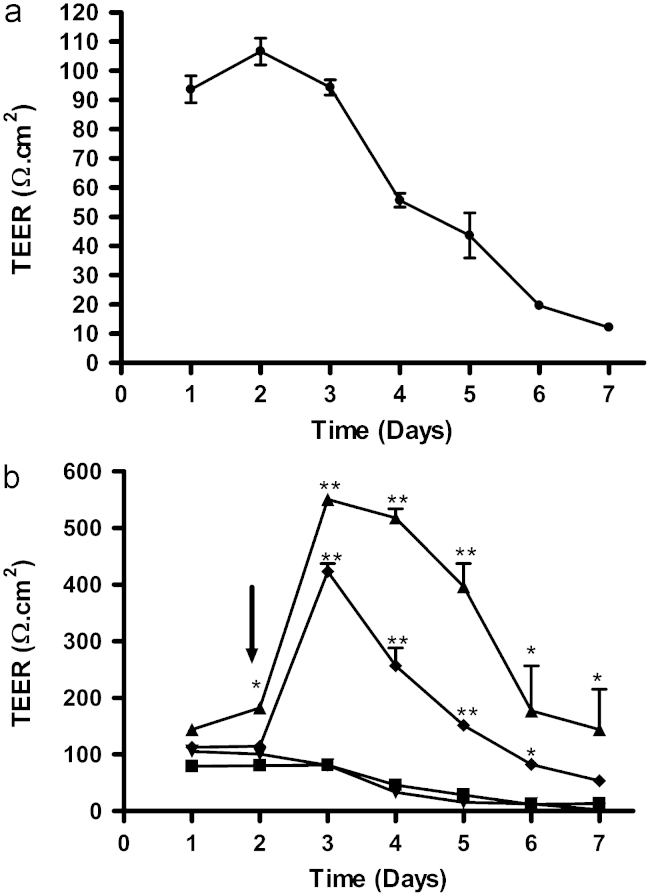
Effect of supplements and serum on TEER in monocultured porcine *in vitro* BBB model (without astrocytes). P.1 PBECs were seeded on Transwell inserts in 12-well plates with normal medium containing serum and the TEER was measured every day for a week (A). In the next series of experiments (B), PBECs were seeded on Transwell inserts, and after 48 h (arrow) the medium was changed to either normal PBEC medium containing serum (▼), PBEC medium minus serum (■), PBEC medium plus supplements (CPT-cAMP, RO 20-1724 and hydrocortisone) (▲) or PBEC medium plus supplements minus serum (♦). Data are means±SEM (*n*=4 Transwell inserts); *p* values are shown for differences from the condition in normal medium, with serum but without supplements (^⁎⁎^*p*<0.01, ^⁎^*p*<0.05; two way ANOVA, followed by Bonferroni *post-hoc* test).

**Fig. 4:**
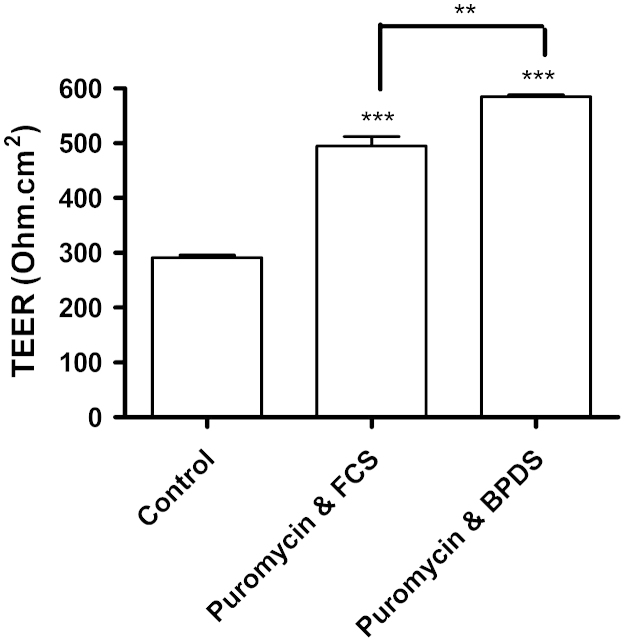
TEER measurements of P.1 PBECs grown using different culture conditions. Cells were grown on 12 mm diameter ‘Transwell Clear’ filter inserts (0.4 μm pore size). Control cells were grown in medium containing foetal calf serum (FCS) without puromycin. Test cells were treated with puromycin and were grown in culture medium containing either FCS or bovine plasma-derived serum (BPDS). Values have been corrected for resistance of a ‘blank’ cell-free insert and are mean±SEM (*n*=3 inserts). Statistical significance was calculated using Student's *t*-test (**p<0.01; ***p<0.001 compared to control).

To optimise the isolation of brain microvessels, special attention was given during initial isolation to removing all meninges (including inside sulci) and most of white matter, and this led to increased culture purity, with fewer contaminating cells growing out from the isolated vessel fragments. The extra time taken over the preparation, while slightly reducing yield, resulted in purer cultures. To reduce the ‘edge effects’ caused by leak of current around the edges of the monolayer at the circumference of the insert, larger inserts were used (12 mm diameter, hence smaller circumference:surface area ratio). In the first series of experiments (Fig. 3A), TEER of cells grown in normal PBEC medium peaked at ∼100 Ω cm^2^ at 2 days and then declined. A similar pattern was seen in cells grown in PBEC medium or medium without serum, but supplementation at 48 h by adding hydrocortisone and increasing cAMP_i_ increased peak resistance to ∼400 Ω cm^2^ in serum-free medium and to ∼530 Ω cm^2^ in serum-containing medium; in supplemented medium, especially medium containing serum, the high resistance phase lasted longer than in normal PBEC medium (Fig. 3B). Puromycin treat-ment was introduced to kill pericytes (Perrière et al., 2005). Addition of 4 µg/mL puromycin in the first three days of growth led to a significant further improvement in purity of the PBEC culture and a significant increase in TEER. In addition, using BPDS rather than foetal calf serum (FCS) in the culture medium also increased TEER (Fig. 4).

#### 2.3.4 Reproducibility and reliability

**Fig. 5:**
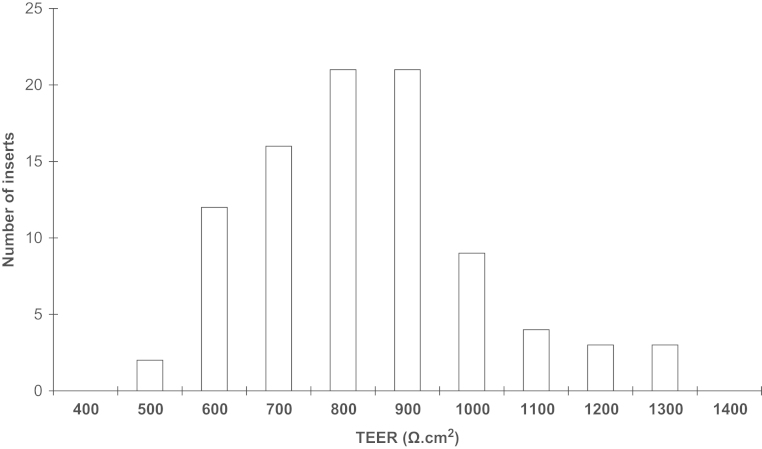
Histogram of TEER data from different PBEC cultures. Puromycin-treated PBECs were passaged and were grown on Transwell inserts for 2 days. Cells were treated with supplements (CPT-cAMP, RO 20-1724 and hydrocortisone) for 24 h and the TEER measured (13 vials from two batches isolated from 12 pig brains). TEER was measured in 91 inserts in 24 independent experiments. TEER of a ‘blank’ cell-free insert has been subtracted from all values.

**Fig. 6:**
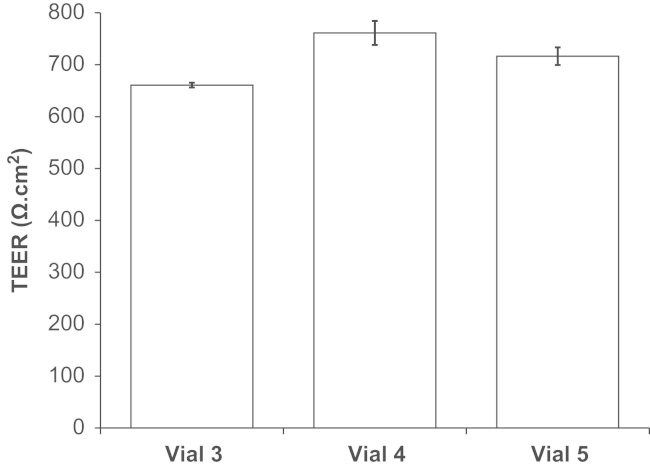
Reproducibility of TEER within a batch of PBECs; P.1 PBECs from different vials. Puromycin-treated PBECs from three vials from the same batch were passaged and grown on 12 mm diameter Transwell Clear filter inserts (0.4 µm pore size) for 2 days. Cells were treated with supplements (CPT-cAMP, RO-20-1724 and hydrocortisone) for 24 h and TEER measured. TEER of a ‘blank’ cell-free insert has been subtracted from all values. Mean±SEM (*n*=3).

**Fig. 7:**
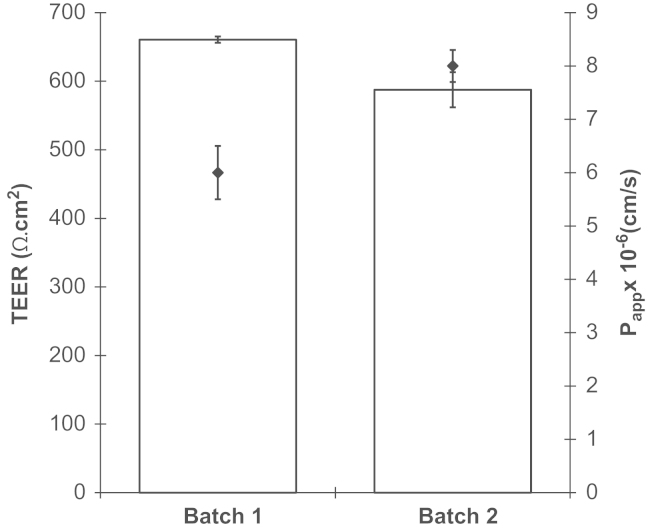
TEER and sucrose permeability of P.1 PBECs from two batches. Puromycin-treated PBECs from two batches were passaged and were grown on Transwell inserts for 2 days. Cells were treated with supplements (CPT-cAMP, RO 20-1724 and hydrocortisone) for 24 h then used for experiments (mean±SEM, *n*=3 inserts per batch). For TEER, the relevant value of a ‘blank’ cell-free insert has been subtracted from all data. TEER is represented by the bars (left *y*-axis) and respective monolayer permeability to [^14^C]sucrose is shown by the points (right *y*-axis).

To reduce variability of TEER observed with the STX2 chopstick electrodes, the WPI Endohm chamber system was used, with large concentric plate electrodes above and below the insert. TEER of 485–1300 Ω cm^2^ (Fig. 5) was typically obtained (mean TEER=789±18 Ω cm^2^; *n*=91 inserts), with good reproducibility between vials (Fig. 6) and batches. Furthermore, the corresponding TEER and *P*_app_ values from each batch confirm the reliability of the model, showing high TEER correlated with low [^14^C]suc-rose permeability (Fig. 7). Mean *P*_app_ for [^14^C]sucrose was 5.7±0.7×10^–6^ cm/s (*n*=7 experiments, 3 inserts each). Further functional characterisation of this phase of the porcine BBB model is described in detail elsewhere (Patabendige et al., this issue).

#### 2.3.5 Co-culture variant

**Fig. 8:**
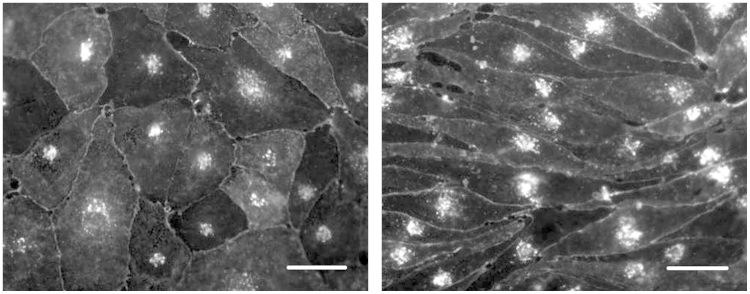
Morphology of P.1 PBECs stained for endothelial cells marker, IB4. P.1 PBECs were seeded on Transwell inserts treated with supplements (cAMP, RO20-1724 and hydrocortisone) and cultured in the absence (A) or presence of astrocytes (B) in the bottom of the well. P.1 PBECs were then treated with IB4-FITC for 30 min, fixed, and mounted for visualisation. Scale bar; 25 µm.

**Fig. 9:**
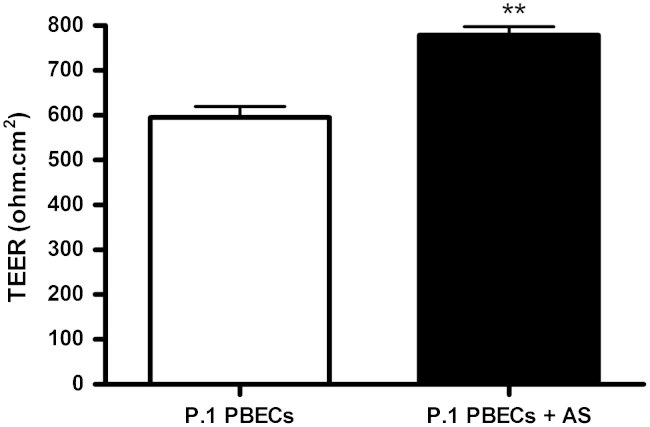
Effect of astrocytes on TEER of porcine *in vitro* BBB model. P.1 PBECs were seeded on Transwell inserts in 12-well plates with (P.1 PBECs+AS) or without (P.1 PBECs) astrocytes (AS) in the bottom of the well. After 48 h, supplements (CPT-cAMP, RO 20-1724 and hydrocortisone) were added and TEER was measured at 72 h. Data are mean±SEM; PBEC, *n*=6 and PBEC +As, *n*=33 Transwell inserts. Statistical significance was calculated using Student's *t*-test (**p<0.01 vs. control PBEC alone).

**Fig. 10:**
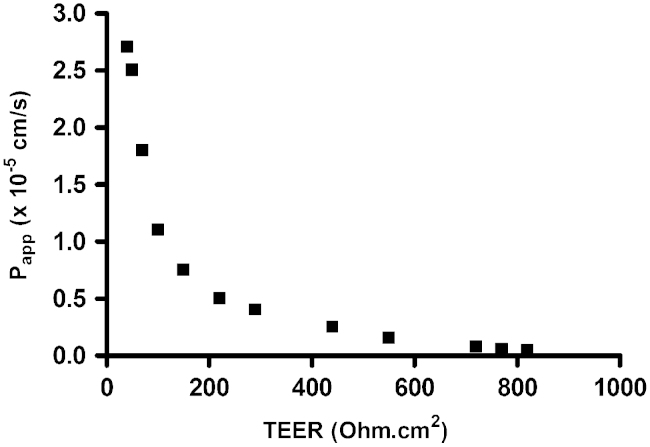
Mannitol permeability of PBEC monolayers as a function of their TEER. P.1 PBECs were seeded on Transwell inserts treated with supplements (CPT-cAMP, RO 20-1724 and hydrocortisone) and cultured in the presence of astrocytes in the bottom of the well. TEER was measured prior to [^14^C]mannitol permeability experiments. Each point represents one Transwell insert.

Pericyte contamination was reduced by differential trypsinisation during passaging the cells before seeding onto inserts and DMEM was used with ACM (i.e. DMEM/ACM). Confluent monocultures of PBECs had an elongated cobblestone-shaped morphology, although not generally so clearly spindle-shaped as reported for rat and bovine brain endothelial cell cultures. However, co-culture of PBECs with astrocytes resulted in a more marked spindle-shaped morphology (Fig. 8). With medium supplemented at 48 h, TEER measured at 72 h was 595±24 Ω cm^2^ in mono-cultured cells, and 779±19 Ω cm^2^ in cells co-cultured with astrocytes in the bottom of the well (Fig. 9). The apparent permeability (*P*_app_) to [^14^C]mannitol measured across the same inserts was in the range 0.1–2.6×10^−5^ cm/s (Fig. 10), and showed an inverse relation to the TEER.

## 3 Discussion

### 3.1 Isolation of microvessel rather than large vessel endothelium

The careful removal of meninges, including its invaginating folds into *sulci*, was designed to remove the large surface vessels, including many of the penetrating arterioles which run perpendicularly into the brain cortex (Dacey and Duling, 1982). This will not only remove most of the potential contamination by leptomeningeal cells with fibroblast-like properties, but also by arterial and arteriolar smooth muscle cells, which tend to grow more rapidly than endothelial cells in culture. The two-stage filtration is designed to retain vessel fragments, allowing isolated cells including most glial cells to pass through. Examination of the material collected from the coarser and finer filters (150 µm and 60 µm mesh respectively) shows that the 150 µm filters retain a less pure (and generally larger diameter) vessel fraction than the 60 µm filters; the latter generate a more homogeneous and higher TEER monolayer consistent with it being derived from relatively pure capillary endothelium. Isolation of predominantly capillary rather than arteriolar or venular microvessels is important as there are several phenotypic and functional differences between the endothelial cells of these different segments of the microvasculature. In particular, compared with arteriolar or venular endothelium, cerebral capillary endothelium has more a more complex and complete pattern of tight junction strands in freeze-fracture images (Nagy et al., 1984) consistent with tighter tight junctions (Wolburg and Lippoldt, 2002), high expression of solute transporters including efflux transporters (Ge et al., 2005; Macdonald et al., 2010; Saubamea et al., 2012), and of certain receptors involved in transcytosis such as transferrin receptor (Ge et al., 2005). Arteriolar endothelium shows higher expression of certain enzymes including 5′-nucleotidase, Mg^2+^-ATPase and Na^+^-K^+^-ATPase than capillary or venular endothelium (Vorbrodt et al., 1982, 1988), and significant absence of P-glycoprotein (Saubamea et al., 2012); bidirectional transcytosis of horseradish peroxidase (creating a local ‘leak’) has been reported in certain brain arterioles but not in capillaries or venules (Westergaard and Brightman, 1973; van Deurs, 1977). The post-capillary venule segment is specialised as a site regulating adhe-sion and traffic of leucocytes into the perivascular space (Bechmann et al., 2007; Owens et al., 2008; Muldoon et al., 2013), shows higher expression of genes involved in inflam-mation-related tasks (Macdonald et al., 2010), and is more affected in inflammatory conditions than capillary endothelium (Paul et al., 2013). Given the much greater area of the cerebral microvascular surface contributed by capillary endothelium compared with arteriolar or venular endothelium (Abbott et al., 2006), preparation of cultures from relatively pure capillary fragments should give the tightest monolayers reflecting most closely the transporting endothelium of the BBB.

### 3.2 Pericyte contamination

In cultures of rat brain endothelial cells, contaminating pericytes frequently grow in the same plane as the endothelial cells, and are typically surrounded by a cell-free zone leading to holes in the endothelial monolayer (Abbott et al., 1992; Parkinson and Hacking, 2005). By contrast, in the porcine model the pericytes generally grow below the endothelial layer, close to or directly on top of the extracellular matrix (see Fig. 2) (Abbott et al., 1997). Hence high TEER can be achieved even in the presence of a small percentage of pericyte contaminants, since they do not necessarily cause holes in the PBEC monolayer. However, PBECs growing on top of pericytes show a slightly altered morphology, with broader cells and irregular cell borders, compared to the elongated spindle-shaped morphology of PBECs without pericyte growth underneath (Fig. 2). In our experience, treatments to remove pericytes as thoroughly as possible gave the tightest monolayers. Puromycin, substrate of the brain drug efflux transporter P-glycoprotein (P-gp) was used to reduce pericytes contamination. Brain endothelial cells have stronger expression of P-gp than pericytes, so can restrict cellular uptake of the cytotoxic puromycin, while pericytes are more vulnerable, tend to be killed by puromycin treatment (Perrière et al., 2005). Proliferating endothelial cells release platelet-derived growth factor (PDGF) that attracts pericytes, and can lead to vessel (tube) formation and release of vascular endo-thelial growth factor (VEGF) through interactions between endothelial cells and pericytes (von Tell et al., 2006). VEGF increases the permeability of the BBB (Dobrogowska et al., 1998). Therefore, reducing the number of pericytes in the culture favours monolayers rather than vessel formation and leads to uniform monolayers of contact-inhibited endothelial cells with low permeability.

### 3.3 Supplementation to increase barrier integrity

Supplementation with treatments to elevate cAMP_i_ was based on a successful protocol for bovine brain endothelial cells (Rubin et al., 1991), and was consistently found to give tighter monolayers in the PBEC model. The treatment of choice now also includes supplementation with hydrocortisone, found to sustain tighter layers in many brain endothelial models (Förster et al., 2008; Hoheisel et al., 1998). In a porcine brain endothelial model developed by Galla and co-workers (Franke et al., 1999, 2000), the presence of ox serum in the medium was found to reduce TEER (Nitz et al., 2003), attributed to the presence of permeabilising factors including lysophosphatidic acid (LPA) and VEGF. Growth fac-tors such as PDGF and VEGF can increase BBB permeability by disrupting tight junctions and stimulating angiogenesis (Dobrogowska et al., 1998; Harhaj et al., 2002; Wang et al., 1996, 2001). To induce better barrier properties, some plasma-derived sera are treated with charcoal to reduce the concentrations of these growth factors. However the charcoal-stripping of serum can lead to removal/reduction of other biologically important factors such as hormones, vitamins, enzymes and electrolytes (Cao et al., 2009). In the present model, we chose to use BPDS, which being derived from adult bovine plasma, is collected with generally less stress to the donor, and contains lower concentrations of growth factors (e.g. PDGF, VEGF) and other vasoactive/proliferative factors than foetal or neonatal calf serum (Abbott et al., 1992). BPDS increased the TEER of the brain endothelial cells compared with serum-free medium, consistent with observations that serum proteins stabilise capillary endothelial permeability, by cross-linking the glycocalyx and possibly also the exposed proteins of the outer zones of the junctional complexes (Curry and Michel, 1980). Where experiments need to be done under serum-free conditions, the monolayers withstand serum removal for 24 h before experiments.

### 3.4 Comparison of variants

Both mono-culture (Patabendige et al., this issue) and co-culture (Skinner et al., 2009) of the PBEC model variants are capable of giving monolayers of TEER >400 Ω cm^2^. For many applications examining the BBB flux of drug-like molecules and other small solutes, this is sufficient to give good resolution between transcellular and paracellular flux (Gaillard and de Boer, 2000). The relationship between *P*_app_ mannitol and TEER observed in our model (Fig. 10) is similar to that reported by Gaillard and de Boer (2000) using two other paracellular permeability markers, sodium fluorescein and 4 kDa FITC-dextran; in our model, *P*_app_ was relatively independent of TEER when TEER was >200 Ω cm^2^. As TEER is inversely related to the small ion conductance (and hence permeability) of the monolayer, TEER recorded at the start of an experiment is a good measure of the ‘basal’ paracellular permeability of the cells, as reference for studies e.g. with drugs which may themselves alter permeability. For leakier monolayers, the TEER can be used to derive a corrected permeability coefficient for a drug from the measured *P*_app_ (Gaillard and de Boer, 2000); however, when TEER is high enough for *P*_app_ to be relatively independent of TEER, the measured *P*_app_ is sufficient without correction, and suitable for comparisons between laboratories.

There is an extensive literature showing that exposure to astrocytes or astrocyte-conditioned medium increases the expression of several BBB features in brain endothelial monolayers (Dehouck et al., 1990; Pottiez et al., 2011) so using the astrocyte co-culture or ACM variants of the method may be required for some applications (Gaillard et al., 2001) including those where vesicular-mediated transcytosis of large molecules is involved (Candela et al., 2008; Demeule et al., 2002; Skinner et al., 2009). However, our experience has been that the state of differentiation of the endothelium also plays a large part in maintaining BBB features, and supplementation with hydrocortisone plus elevation of cAMP_i_, combined with growth on extracellular matrix mimicking the native brain endothelial/astrocyte basement membrane, without addition of astrocyte-derived influence, may be sufficient for many applications.

### 3.5 Comparison with PBEC models in the literature

Several promising PBEC models have been introduced over the last decade (Cohen-Kashi Malina et al., 2009; Franke et al., 2000; Smith et al., 2007; Zhang et al., 2006). However, several things drove our development of an alternative to published methods. At the start of this process (early 1990s) there was no reliable published method for generating porcine brain endothelial cells. Since then, several methods have been described, but intra-batch and batch-to-batch variation was still a problem with many of them (Franke et al., 2000; Zhang et al., 2006). There was some variability in the effects of adding serum, reported to either increase or decrease permeability (Nitz et al., 2003), and it was not always clear whether astrocytic influence was necessary. While astrocytes were not required to generate a high TEER in the PBEC model described by Franke et al. (2000), others have reported that astrocytic influence is necessary to produce a practical model (Cohen-Kashi Malina et al., 2009; Smith et al., 2007). In general, where a brain endothelial cell culture model achieves a high TEER without astrocytic influence (Franke et al., 2000; Lohmann et al., 2002; Patabendige et al., this issue; Zhang et al., 2006), functional expression of small solute transporters (SLCs) and efflux transporters is found to be sufficient to allow use of the monocultures for drug permeability assay. For leakier models, co-culture with astrocytes (Cohen-Kashi Malina et al., 2009; 2012; Kido et al., 2002) or C6 glioma cells, or exposure to glial-conditioned medium (Smith et al., 2007) may be necessary to tighten the barrier and improve expression of other BBB properties such as enzymes and transporters, to produce functional assay systems. For certain specialised features of the brain endothelium such as receptor-mediated transcytosis, astrocyte co-culture may be necessary even with tighter monolayers (Skinner et al., 2009).

A detailed comparison of the methods and barrier characteristics of the main PBECs models in comparison to our model is given in Patabendige et al. (this issue). The strengths of the present method are that it is relatively simple, involving fewer preparative steps, and that it gives a high yield. With this method (Patabendige et al., this issue), reliably tight brain endothelial cell monolayers can be grown on inserts without astrocyte influence, and if needed, serum can be removed for the last 24 h to provide suitable starting conditions for experiments, without significantly compromising tightness. The alternative co-culture variant of this method described provides considerable flexibility for experimental design, depending on the application.

The ultimate goal for most BBB researchers is to be able to study the human BBB. However, the difficulties associated with developing robust and realistic *in vitro* human BBB models have led to the use of animal models (Patabendige, 2012). A porcine BBB model is a good alternative as the biology of the pig is closer than that of other laboratory animals to the biology of the human (Walters et al., 2011). The PBEC model presented in this paper is one of the best BBB models giving high TEER. However, as with all BBB models, there are some limitations. Strict adherence to the experimental procedure is required to produce high yields of pure PBEC cultures and to minimise variation between batches. Only limited *in vivo* data is available for porcine models compared to rodent models; however, with the increased use of transgenic and miniature pigs this will improve in future. Availability of good porcine primers and antibodies is currently an issue, but this also will improve with the recent publication of a high-quality draft pig genome sequence (Groenen et al., 2012). Further examination of expression and function of transporters and receptors on the PBEC model is currently under way.

In summary, this method combines simplicity and reproducibility with optimum cell yield and purity, making the resulting PBEC model robust, reliable and flexible, with good preservation of BBB features, suitable for a range of appli-cations.

## 4 Experimental procedure

### 4.1 Time required

8 h isolation of brain capillaries and freezing (from 6 pig brains)
3 days cell culture
1 h passaging of cells onto Transwells filter inserts
2–3 days cell culture to confluence
15 min medium exchange (‘switch medium’)
24 h later: ready for experiments
The total time required from cell preparation to having suitable cell cultures for mechanistic and transport studies is 8 days, but the cells stay in well-differentiated state for up to 4 days after this.

### 4.2 Materials

Culture medium L-15 Leibovitz (L-15); medium 199 (M199); DMEM; Penicillin (10,000 U/mL)/Streptomycin (10 mg/mL) (P/S); Glutamine (2 mM stock soln); Heparin; Puromycin; cell permeant cAMP analogue, CPT-cAMP; Hydrocortisone; Trypsin-EDTA for endothelial cells; Hanks’ balanced salt solution (HBSS) without (w/o) Ca^2+^,Mg^2+^; FCS; poly-D-lysine; human fibronectin; dimethyl sulfoxide (DMSO); all from Sigma. Type IV phosphodiesterase inhibitor, RO 20-1724 from Calbiochem/Merck. Enzymes from Lorne Laboratories Limited, UK. Collagenase, Trypsin, DNase I. Minimal essential medium (MEM+HEPES) from MP Biomedicals. Phosphate buffered saline (PBS) with Ca^2+^ and Mg^2+^ from Cambrex Bio Science. BPDS from First Link UK. Nylon meshes (60 µm and 150 µm pore size) from Plastok Associates, UK. Rat tail collagen type I from Becton Dickinson. Tissue culture plastics (flasks, plates, Petri dishes). Filter inserts: Costar ‘Transwell Clear’ 12-well tissue-culture-treated sterile polyester membrane, 0.4 µm pore, 12 mm membrane, pre-loaded on cluster plates. [^14^C]sucrose (0.15 µCi/mL final concentration, specific activity 643 mCi/mmol) and [^14^C]mannitol (0.20 µCi/mL final concentration, specific activity 56 mCi/mmol) from GE Healthcare. Anti-smooth muscle-specific actin (monoclonal-mouse) from Dako Ltd.; monoclonal antibody against p100/120 from Transduction Laboratories (now BD Biosciences). Anti-mouse secondary antibodies were from Jackson Immunoresearch Laboratories Inc. and nuclear stain Hoechst 33342 was from Sigma. FITC-labelled IB4 was from Gibco, Paisley, UK and ProLong Mounting Medium containing Dapi was from Invitrogen, UK. Lab-made rat-tail collagen (Strom and Michalopoulos, 1982). All other chemicals not quoted specifically were obtained from commercial sources at the highest quality available.

### 4.3 Laboratory equipment

• Refrigerated centrifuge
• Dounce glass homogeniser (40 mL, with loose and tight pestle)
• Dissecting instruments set: fine forceps (for fine dissection, removal of meninges), curved forceps (for separation of white matter from grey matter), coarse forceps (‘rat-toothed’), scalpel
• Nalgene reusable filter holder unit for 47 mm diameter membranes (500 mL receiver) from Fisher Scientific
• Three clean glass beakers
• Sterile plastics: 1 L containers, 15 cm Petri dishes, 50 mL centrifuge tubes, 50 mL syringe, T75 and T175 flasks, sin-gle-wrapped tissue culture pipettes (10 mL, 25 mL)
• 200 mL glass bottle for preparation of ‘Digest Mix’ stock
• 60 µm and 150 µm nylon mesh, cut slightly larger than the size of filter unit; gauze and paper towels
• Measurement of TEER: EVOM2 voltohmeter with ENDOHM-12 electrode chamber, and/or STX2 chopstick electrodes, both from World Precision Instruments, USA
• Automatic pipettes+tips (e.g. Gilson)

### 4.4 Composition of solutions and media

• *Transport solution* for transferring brains to laboratory. L15 medium with added penicillin (100 U/mL), streptomycin (100 µg/mL) (Pen/Strep).
• *Washing and dissection solution*: HEPES buffered MEM containing 10% FCS and 1% P/S (% by volume).
• *Digest Mix***:** *Enzymes in M199 medium with FCS and P/S*. Weigh out 188 mg collagenase (223 U/mg), 86 mg trypsin (211 U/mg), 10.9 mg DNase I (2108 U/mg). Add 178 mL of M199 and filter sterilise into an autoclaved 200 mL glass bottle. Then add 20 mL FCS and 2 mL P/S. Aliquot into centrifuge tubes and freeze at −20 °C.
• *Freezing mix*: Resuspend vessel fragments in 10% DMSO in FCS and aliquot to cryovials (1 mL each), bring slowly to −80 °C (use a cryo freezing container to achieve −1 °C/min cooling rate; place vials in the container and place it in −80 °C freezer for 24 h), then store vials in liquid nitrogen.
• *Basic growth medium*: DMEM, 10% BPDS, 1% P/S, 1% Glutamine and 125 μg/mL heparin. Pass through 0.22 µm filter before use.
• *Switch* (*differentiation*) *medium*: Once cells are growing well on inserts, change growth medium to serum-free switch medium containing DMEM, 1% P/S, 1% Glutamine, 125 μg/mL heparin and 550 nM hydrocortisone. Then treat cells with 250 µm CPT-cAMP and 17.5 µm RO 20-1724 (see below for timings).

### 4.5 Animals and yield

The culture of each batch of cells starts with six pig brains (from abattoir), and generates 12 cryovials each of ‘60s’ and ‘150s’, indicating the filter mesh size used for their isolation. One vial is sufficient for two T75 flasks and cells from two T75 flasks are enough for 18–24 Transwell 12 mm diameter inserts (1×10^5^ cells/insert). Hence six brains yield ∼24×20=480 Transwell inserts with confluent cells.

### 4.6 Preparations (day before isolation)

• Sterilise dissecting instruments, glass beakers, homogeniser, filter unit, six circles each of 60 µm and 150 µm nylon mesh, gauze and sterile 1 L containers
• Prepare a solution of L-15 medium with 1% P/S (L-15+)
• Prepare a solution of PBS with Ca^2+^ and Mg^2+^ with 1% P/S (PBS+)
• Prepare a solution of MEM/HEPES medium with 10% FCS and 1% P/S (Mem/HEPES+)
• *Sterile disposables*: Scalpel, cell scrapers, 50 mL syringes, single-wrapped tissue culture pipettes, Petri dishes, centrifuge tubes, labelled cryovials, T75 and T175 flasks

### 4.7 Detailed experimental procedure

#### 4.7.1 Isolation of brain capillaries

1. *Collect brains from abattoir*: Acquire 12 fresh porcine brain hemispheres from the abattoir. Wash each hemisphere briefly in L-15+ and transport brains to lab in three sterile 1-litre tubs containing L-15+ on ice.
2. *Wash brain*: Pour a little PBS+ into a beaker. Remove one hemisphere from the container and wash thoroughly in PBS+.
3. *Remove meninges*: Place some gauze in a Petri dish. Place one hemisphere in your hand directly above the Petri dish. Using fine forceps carefully remove the meninges. Make sure to remove the meninges inside the grooves (sulci). Wash again and place in a new beaker containing fresh PBS+. Spray hands with 70% ethanol. Repeat for all the hemispheres.
4. *Remove white matter*: Place a cleaned hemisphere in your hand. Remove the white matter (cut off large chunks where possible, and use curved forceps to pinch off small whole sections of white matter). Place grey matter in a beaker containing MEM/HEPES+. Repeat for all hem-ispheres.
5. *Cut into small pieces*: Remove the MEM/HEPES+ in beaker and replace with fresh MEM/HEPES+. Use a scalpel to chop the brain into pieces of ∼1 cm^3^.
6. *Extrude brain material through syringe*: Half fill a 50 mL syringe with the brain matter (use forceps to transfer) and squeeze it into a T75 flask containing 50 mL MEM/HEPES+.
7. *Homogenise*: Pour 15 mL of brain extract from the T75 into the homogeniser. Top up with MEM/HEPES+ to just below the wider part of the homogeniser (total volume should be about 40 mL). Homogenise very gently up and down for 15 strokes with the loose pestle and then 15 strokes with the tight pestle. Pour homogenate into a clean T175. Continue until all tissue has been homogenised, pool homogenate (1000–1200 mL).
8. *First filtration using 150 µm mesh*: Filter about 200 mL of the homogenate through a sterile 150 μm mesh. Rinse the filter with about 40 mL of MEM/HEPES+. Remove filter from the filter unit and place the filter in a 15 cm Petri dish containing 80 mL of digest mix. Continue with further filters, adding them to the dish. Incubate filters for one hour at 37 °C on a shaker or rocker. This will make the ‘150 s’ sample.
9. *Second filtration using 60 µm mesh*: Take filtrate from step 8 and filter again, this time through 60 μm mesh (200 mL per filter). Again rinse and remove each filter and add to a second Petri dish containing 80 mL of digest mix. Incubate for one hour at 37 °C on an orbital shaker or rocker. This will make the ‘60s’ sample.
10. *Harvest microvessels from filters, keep 150s and 60s separate*: Collect the capillaries bound to the 150 µm filters using a pipette. Remove the digest mix and place into two 50 mL centrifuge tubes and spin down for 5 min at 240g at 4 °C. Repeat for 60 µm filters, and from this point on keep the resulting sample separate from the ‘150s’. These fractions generally result in different qualities of endothelial cultures—cells derived from capillaries caught on the 60 µm filters usually having fewer contaminating peri-cytes.
11. *Triturate microvessel fractions*: Aspirate off the supernatant from the 150s and resuspend pellet in 10 mL of MEM/HEPES+. Triturate suspension. Add 20 mL of MEM/HEPES+ and triturate up and down. Centrifuge again for 5 min at 240*g* at 4 °C. Repeat for the 60s.
12. *Resuspend and spin again*: Repeat step 11 (i.e. resuspend each pellet in MEM/HEPES+ and spin again for 5 min at 240*g* at 4 °C).
13. *Transfer cells into freezing mix*: If cells are not being used immediately, resuspend each pellet (150 s and 60 s) in 10.8 mL of FCS and 1.2 mL DMSO. Pipette 1 mL aliquots into labelled cryo-vials. Transfer the vials into the freezing container and freeze at −80 °C overnight. Then store in liquid nitrogen.

#### 4.7.2 Thawing and growth

Coat two T75 flasks with lab-made rat tail collagen (300 µg/mL in sterile water) for 2 h at RT. Remove collagen and wash twice with HBSS and add fibronectin (7.5 µg/mL in sterile water) and leave for 2 h at RT. After two hours remove fibronectin and wash twice with HBSS. Alternatively, flasks can be coated with rat-tail collagen only for 3 h at 37 °C. Thaw one aliquot per two collagen/fibronectin-coated T75 flasks. Thaw vials by immersing the bottom half of the cryovial in a water bath (37 °C) for 2–3 min, swirling gently. Add the thawed aliquot to 16 mL of basic growth medium (containing 4 µg/mL puromycin) and pipette into flasks. PBECs become ∼80% confluent within 3 days and can be passaged at this stage.

#### 4.7.3 Setting up PBEC mono-culture model on Transwell filters

Rinse cells twice with HBSS without Ca^2+^, Mg^2+^. Add 2 mL of trypsin-EDTA per flask and put flask back into the incubator for 3–5 min and then continually observe under the microscope. Shake the flask to detach endothelial cells and tap gently if necessary. When the majority of endothelial cells have come off add 8 mL of basic growth medium (without puromycin) and transfer the contents of the flask to a centrifuge tube. Spin the cells for 5 min at 380*g*. Resuspend the pellet in 1 mL of medium, count cells and seed the passaged PBECs onto Transwell inserts at 1.0×10^5^ cells/cm^2^. Use basic growth medium without puromycin until P.1 PBECs become 100% confluent.

#### 4.7.4 Special treatments to induce differentiation (‘Switch’ medium)

P.1 PBECs become confluent 2–3 days after passaging onto inserts. When 100% confluent, change the medium to serum-free switch medium and treat with 250 µM CPT-cAMP and 17.5 µM RO 20-1724. P.1 PBECs are ready for experiments after 24 h of this treatment. 60s give the best endothelial cells (uniform, derived from smaller vessels) and should be used for Transwell experiments; TEER range: 400–1300 Ω cm^2^. 150s can be used for immunostaining and RNA/protein isolations; still give a high percentage of endothelial cells but are more likely to be from larger vessels and therefore, may have more contaminating cells. TEER range: 100–400 Ω cm^2^; can be higher if grown for longer.

### 4.8 Co-culture variant

#### 4.8.1 Astrocyte isolation

Prepare primary cultures of rat astrocytes by the method described by McCarthy and de Vellis (1980). In brief, dissect out cortices from 0 to 2-day-old Sprague-Dawley rat pups, remove meninges and dissociate through a nylon net. Collect the filtrate, centrifuge for 10 min at 200*g* and re-suspend the pellet in 10 mL DMEM with 10% FCS and 1% P/S. Seed at 5×10^5^ cells/mL in poly-D-lysine coated T75 flasks and incubate for 5 days. Change the medium every 3 days until 100% confluent. Remove cell contaminants by shaking on an orbital shaking system at 37 °C overnight. Dissociate astrocytes using trypsin, centrifuge cells for 5 min at 200*g* and re-suspend the pellet in DMEM with 10% FCS and 1% P/S. Seed at 1×10^5^ cells/mL into poly-D-lysine coated-12-well plates and culture for 10 days. Determine purity (over 95%) by glial fibrillary acidic protein expression.

#### 4.8.2 Astrocyte-conditioned medium

For collection of ACM, feed astrocyte cultures with fresh DMEM containing 10% BPDS. After 48 h, filter the conditioned medium through a 0.2 µm pore nitrocellulose membrane to remove cell fragments, snap freeze in dry ice and store at −80 °C.

#### 4.8.3 Setting up PBEC co-culture model

Add a thawed PBEC aliquot to 36 mL of basic growth medium (without puromycin) and pipette into collagen/fibronectin-coated 6-well plates. After 4 h, change the medium to 50% ACM, 50% basic growth medium. PBECs should be passaged when ∼60–70% confluent.

Rinse cells with PBS and then with warm EDTA/PBS. Add trypsin and put plate back into the incubator for 2 min and then continually observe under the microscope. The endothelial cells are more sensitive to trypsin so will come off first. Shake the plate gently but do not tap; tapping will cause the cells to be removed in sheets taking the pericytes with them. When the majority of endothelial cells have come off, transfer the contents of the plate to a centrifuge tube con-taining 0.5 mL FCS. Spin the cells for 5 min at 240*g*. Resuspend the pellet in 1 mL of basic growth medium, count cells and seed onto Transwell inserts at 8×10^4^ cells/insert. Transfer the inserts to a 12-well plate containing confluent rat astrocytes. Change the medium to ‘Switch’ medium when PBECs are 100% confluent.

### 4.9 Assessing barrier integrity and reproducibility: Measuring TEER and mannitol or sucrose permeability

#### 4.9.1 TEER measurements

BBB integrity can be assessed non-invasively and in real time by TEER measurement. TEER was recorded in an Endohm chamber or with STX2 chopstick electrodes (co-culture variant) connected to an EVOM resistance meter (World Precision Instruments, Sarasota, FL, USA). According to Ohm's law, *V*=*IR* where *V*, voltage; *I*, current; *R*, resistance. Resistance is inversely proportional to permeability (or conductance), and reflects permeability to small ions carrying electrical current. For Endohm, PBECs grown on Transwell inserts were placed between the flat plate silver–silver chloride electrodes. When chopstick electrodes were used, they were placed at a uniform distance from the cells grown on the inserts. Control resistance measurements from ‘blank’ cell-free inserts were subtracted to calculate the resistance of the cell monolayer. Resistance values were multiplied by the surface area of the insert membrane to express results in Ω cm^2^.

#### 4.9.2 Permeability studies

[^14^C]sucrose permeability studies were performed on cell monolayers with TEER>500 Ω cm^2^. Culture medium was aspirated off the inserts and the inserts were transferred to 12-well plates (placed in a shaker at 37 °C) containing 1.5 mL/well of assay buffer (DMEM without phenol red, 25 mM HEPES and 0.1% bovine serum albumin). 0.5 mL of assay buffer containing [^14^C]sucrose (final concentration: 0.15 µCi/mL was added to the first insert and then to other inserts at 10-s intervals. At *t*=5 min, the inserts were transferred to the next well containing assay buffer. This procedure was repeated for all inserts at *t*=15 min and 30 min. At the end of the experiment (*t*=30 min), samples were taken from each insert (50 µl sample+150 µl of assay buffer) and well (200 µl sample) to scintillation vials, 5 mL of scintillation fluid added, and vials counted in a scintillation counter. For the co-culture variant, permeability studies were performed using [^14^C]mannitol on cells grown to confluence on Transwell inserts with a minimum TEER of 250 Ω cm^2^. [^14^C]mannitol was added to the insert (final concentration 3.6 μM). Samples (100 μL) were taken from the well after 0, 1 and 3 h. The samples were added to 1 mL of scintillation fluid and counted in a scintillation counter.

Cleared volume was plotted as a function of time and the slope was obtained by linear regression. The slope of the clearance curve represents the PS product (permeability×surface area). Apparent permeability (*P*_app_, cm/s) was calculated by dividing the PS product by the surface area of the filter.

### 4.10 Immunocytochemistry and cell staining

#### 4.10.1 Immunocytochemistry

Transwell inserts were fixed in 4% paraformaldehyde for 10 min, washed in PBS, permeablised in 0.5% Triton-X-100 in PBS for 20 min then blocked for 30 min in 10% calf serum with 0.1 M lysine and 0.3% Triton-X-100 in PBS. Primary antibodies were added in blocking solution at 4 °C overnight. Transwell inserts were then washed and secondary antibodies added in blocking solution with added nuclear stain Hoechst 33342 at 1 µg/mL for 1 h at room temperature.

#### 4.10.2 Cell staining using FITC labelled IB4

Cells were cultured on Transwell inserts and FITC-labelled IB4 (1:200 dilution) was added to the apical side for 30 min in the dark. The cells were then washed with PBS, fixed with 4% paraformaldehyde and mounted using Pro-Long Mounting Media containing Dapi. Images of the stained cells were obtained using a fluorescent microscope attached to a digital camera.

### 4.11 Statistical analysis

Data are expressed as mean (±standard error of the mean, SEM) and analysed and presented using GraphPad Prism. Groups of two were analysed using Student's *t*-test, groups of three or more were analysed using either one-way analysis of variance (ANOVA) with a Dunnets *post-hoc* test or, if multiple variables were involved, two-way ANOVA with Bonferroni *post-hoc* test was applied. Values were considered to be significantly different when *p*<0.05.
